# Sex differences in the role of AKAP12 in behavioral function of middle-aged mice

**DOI:** 10.1186/s13293-024-00670-8

**Published:** 2024-11-21

**Authors:** Hidehiro Ishikawa, Shintaro Kimura, Hajime Takase, Maximillian Borlongan, Norito Fukuda, Tomonori Hoshino, Gen Hamanaka, Ji Hyun Park, Akihiro Shindo, Kyu-Won Kim, Irwin H. Gelman, Josephine Lok, Eng H. Lo, Ken Arai

**Affiliations:** 1 Neuroprotection Research Laboratories, Departments of Radiology and Neurology, Massachusetts General Hospital and Harvard Medical School, 149 Thirteenth Street, Room 2401, Charlestown, MA 02129-2000 USA; 2 Department of Neurology, Mie University Graduate School of Medicine, 2-174 Edobashi, Tsu, Mie Japan; 3 Life Science Research Center, Gifu University, Gifu, Japan; 4 YCU Center for Novel and Exploratory Clinical Trials (Y-NEXT), Yokohama City University Hospital, Yokohama, Japan; 5 College of Pharmacy and Research Institute of Pharmaceutical Sciences, Seoul National University, Seoul, 08826 Republic of Korea; 6 Department of Cancer Genetics and Genomics, Roswell Park Comprehensive Cancer Center, Buffalo, NY USA; 7 Pediatric Critical Care Medicine, Department of Pediatrics, Massachusetts General Hospital and Harvard Medical School, Boston, MA USA

**Keywords:** Age, AKAP12, Behavior, Knockout mice, Mouse, Sex difference

## Abstract

A-kinase anchoring protein 12 (AKAP12) is a key scaffolding protein that regulates cellular signaling by anchoring protein kinase A (PKA) and other signaling molecules. While recent studies suggest an important role for AKAP12 in the brain, including cognitive functions, its role in middle-aged mice and potential sex differences are not fully understood. Therefore, this study investigated the effects of AKAP12 on cognitive and exploratory behavior in middle-aged mice, focusing on sex differences. Cognitive function was assessed using the spontaneous Y-maze test and the novel object recognition test (NORT). No significant sex differences in cognitive function were found in middle-aged C57BL/6J mice; however, female mice showed greater exploratory behavior during the NORT. In addition, both middle-aged male and female Akap12 knockout (KO) mice performed similarly to wild-type (WT) mice in the Y-maze test, but had lower discrimination indices in the NORT, suggesting a potential role for AKAP12 in short-term memory. Notably, exploratory behavior was suppressed in female Akap12 KO mice compared to WT mice, whereas male Akap12 KO mice did not show this effect. There were no significant differences in movement distance and velocity during the Y-maze test and NORT between WT and KO mice of either sex. These results indicate that AKAP12 affects cognitive function and exploratory behavior in middle-aged mice and that these effects differ between sexes.

**Supplementary Information:**

The online version contains supplementary material available at 10.1186/s13293-024-00670-8.

## Highlights

This study examines the role of A-kinase anchoring protein 12 (AKAP12) in cognitive and exploratory behavior, focusing on sex-related differences in middle-aged mice. Our results show that AKAP12 deficiency impairs short-term memory in both sexes, but specifically reduces exploratory behavior in female mice, highlighting a potential sex-specific role of AKAP12 in regulating exploratory behavior. Given the growing interest in understanding the molecular and cellular mechanisms underlying brain function and behavior, particularly in the context of sex differences, our findings will provide valuable insights into the field of neuroscience.

**Supplementary Information:**

The online version contains supplementary material available at 10.1186/s13293-024-00670-8.

## Abstract (fr)

This study examines how a protein called A-kinase anchoring protein 12 (AKAP12) affects memory and behavior in middle-aged mice, and whether it works differently in males and females. AKAP12 is known to regulate important brain functions, but its role in potential sex differences hasn’t been fully explored. In this study, we tested the memory and exploratory behavior of the mice. Both male and female mice performed similarly in memory tests, but female mice explored objects more than males. When AKAP12 was removed from the brains of the middle-aged mice, both males and females showed memory problems, but only females had reduced exploratory behavior. Our results suggest that AKAP12 is important for memory maintenance and influences behavior in middle-aged mice. In addition, the differences we observed between males and females highlight the potential of AKAP12 as a target for treatments aimed at memory and cognitive problems, especially as people age or develop brain-related diseases.

## Introduction

A-kinase anchoring proteins (AKAPs) are a family of scaffolding proteins that includes more than 50 variants. AKAPs are known for their ability to anchor protein kinase A (PKA) and other signaling proteins to specific subcellular locations, such as the nucleus, plasma membrane, or mitochondria, and play a key role in the spatial and temporal regulation of cellular signaling pathways. AKAP-based signaling complexes coordinate cAMP signaling with other signaling pathways, leading to a wide range of cellular functions. There is increasing evidence that some AKAPs have physiological functions in the central nervous system (CNS) [1, 2]. For example, AKAP1 is involved in maintaining mitochondrial trafficking within the dendrites of cortical neurons by scaffolding PKA to phosphorylate Miro-2, thereby enhancing dendritic sprouting [3]. In addition, AKAP5 has been reported to promote synaptic plasticity by recruiting PKA, calcineurin, and protein phosphatase 2B to phosphorylate GluA1/1 and CP-AMPAR, which are critical for synaptic strength and memory formation [4]. AKAP12 (also known as SSeKS or Gravin) is also expressed in the CNS and plays a role in CNS function [5, 6]. AKAP12 is predominantly cytoplasmic and translocates to the plasma membrane during neuronal differentiation. It colocalizes with signaling complexes such as protein kinase C (PKC) and protein phosphatase 2B [7] and translocates to the perinuclear region in differentiated neurons following PKC-induced phosphorylation [8]. However, how AKAP12 contributes to brain function still remains to be elucidated.

Different expression patterns of AKAP12 have been observed in the brain. For example, expression patterns in the cerebellum and brainstem differ from those in other brain regions, with elevated levels in 4-week-old rats [9]. In addition, another study showed that AKAP12 expression levels were higher in the corpus callosum compared to the cerebral cortex in both 2- and 8-month-old mice [10]. Interestingly, AKAP12 expression in the brain is age-dependent, with higher levels during early developmental stages that gradually decrease with age [9]. This phenomenon may support the idea that AKAP12 contributes to cognitive function, as aging is a major risk factor for cognitive decline. In fact, AKAP12 deficiency caused impaired hippocampus-independent spatial memory without affecting motor function [11]. This study also showed long-term memory impairments in the object recognition test and the fear conditioning test in Akap12 knockout (KO) mice [11]. In addition, our group showed that working memory was impaired in Akap12 KO mice using the Y-maze test [12]. However, these studies primarily used young male mice, and there is a gap in knowledge regarding the role of AKAP12 in female and/or middle-aged mice. Therefore, in this study, we aimed to investigate whether there is a sex difference in the role of AKAP12 in behavioral function in middle-aged mice.

## Method

### Animals

All experimental procedures followed NIH guidelines and received approval from Massachusetts General Hospital Institutional Animal Care and Use Committee. C57BL/6J mice were purchased from The Jackson Laboratory (ME, USA). Akap12 KO mice, on a C57BL/6J background, were provided from the Gelman Lab at Roswell Park Comprehensive Cancer Center [13] and subsequently maintained and expanded at the animal facility of Massachusetts General Hospital. The mice were housed in a specific pathogen-free conditioned 12-h light/dark cycle room (lights on at 7am and off at 7pm) with free access to food and water throughout the experiment. The protocols for genotyping Akap12 KO mice lines were conducted as previously described [12]. The representative genotyping PCR image and qPCR data from samples of randomly selected mice used in this study are provided in Supplementary Figure S1.

### Western blot

Male and female C57BL/6J mice were used for the western blot experiments. Brains were removed following transcardial perfusion with ice-cold 0.1 M phosphate-buffered saline (PBS) at a volume of 2 mL/g body weight. Tissue samples of cerebral frontal cortex, corpus callosum, and hippocampus (2- and 12-month-old, males and females, *n* = 10 each) were dissected in NP40 cell lysis buffer (MyBiosource, CA, USA) [10]. After centrifugation and aspiration of the supernatant, the samples were digested with equal volumes of LDS sample buffer containing a reducing agent (Thermo Fisher Scientific, MA, USA) and heated at 70 °C for 10 min. Seven micrograms of each sample were loaded onto 4–12% Bis-Tris gels. Following electrophoresis and transfer to nitrocellulose membranes, the membranes were blocked with 5% skim milk in Tris-buffered saline (TBS) containing 0.1% Tween-20. Subsequently, the membranes were incubated with a primary antibody against AKAP12 (1:1,000, obtained from the Gelman Lab) and β-actin (1:10,000, A5441, Sigma-Aldrich, MO, USA). The membranes were then processed with peroxidase-conjugated secondary antibodies (1:2,000, Jackson Immunoresearch Laboratories, PA, USA) and visualizing using Pierce ECL Western Blotting Substrate or SuperSignal™ West Pico PLUS Chemiluminescent Substrate (Thermo Fisher Scientific, MA, USA). Visualized bands were semi-quantified using ImageJ by an operator who was blinded to the group allocation. We used samples from whole brains of 2-month-old male mouse as a control for western blot experiments.

### Y-maze test

Experiments were conducted between 7am and 10am to assess working memory using the 8-minitue spontaneous Y-maze alternation test [12, 14]. These WT and KO mice used in the study were middle-aged (12–14 months old), and mice that were used for the Y-maze test were also used for the NORT experiments, but not for the western blot experiments. The maze consists of three arms, each 40 cm long, 9.5 cm high, and 4 cm wide, labeled arm-A, -B, or -C, diverging at 120° from the central point (Muromachi Kikai, Tokyo, Japan). Each mouse was placed in a Y-maze and allowed to move freely through the maze during 8-minute session. This task was videotaped using a camera and the sequence of arm entries was manually recorded in a blinded manner. To assess the locomotor activity of the mice, we measured the velocity and total distance traveled. Movement distance and mean velocity during the Y-maze test were calculated using EthoVision XT V17 (Noldus Information Technology, Wageningen, The Netherlands). An actual alternation was defined as entries into all three arms on three consecutive runs. The maximum alternation was calculated by subtracting two from the total number of arm entries. The percentage of alternation was then calculated using the formula: (actual alternation / maximum alternation) × 100%. The total number of arm entries during the session was recorded to assess locomotor activity. All experiments and analyses were conducted by an investigator who was blinded to the group allocation.

### Novel object recognition test (NORT)

Experiments were conducted between 7am and 10am to assess short term recognition memory using the NORT [15–18]. These WT and KO mice used in the study were middle-aged (12–14 months old), and mice that were used for the NORT were also used for the Y-maze test, but not for the western blot experiments. In the NORT, mice were first placed in a clean, empty cage for 10 min. Subsequently, they were exposed to two identical objects in the same cage for 5 min (acquisition period). After 30-minute interval, mice were presented with two different objects (one original and one novel object, placed in the same positions as during the acquisition period) in the same cage for 5 min (retention period). Object recognition was videotaped and scored based on the total investigation time, defined as either sniffing or touching the object. To assess the locomotor activity of the mice, we measured the velocity and total distance traveled. Movement distance and mean velocity during the retention phase were calculated using EthoVision XT V17 (Noldus Information Technology, Wageningen, The Netherlands). The delta exploration time was determined by subtracting the total time spent in the acquisition phase from the total time spent in the retention phase. To follow our previous studies [15, 19], short-term recognition memory performance was quantified by the discrimination index, calculated using the formula: DI = (the time spent on the novel object) / (the total time spent on both objects) – 0.5 (ranging from − 0.5 to 0.5). There are two reasons for using this formula; (i) To establish a neutral baseline: Subtracting 0.5 adjusts the DI to a range between − 0.5 and 0.5. A score of zero indicates that equal time was spent on the novel and familiar objects, while a positive score indicates more time was spent on the novel object, and a negative score indicates a preference for the familiar object. (ii) Symmetrical scoring: By adjusting the score to range between − 0.5 and 0.5, the index becomes symmetric, making it easier to compare results across groups. All experiments and analyses were conducted by an investigator who was blinded to the group allocation.

### Statistical analysis

Statistical analyses were performed with Prism 9.4.1 (GraphPad, Software Inc., USA). Statistical significance was evaluated using Mann-Whitney test or Unpaired t-test for 2 group comparisons or 2-way ANOVA for multiple comparisons, as appropriate. Normality of the data was assessed using the Shapiro-Wilk test. Data are expressed as mean plus and/or minus S.D. A p value of < 0.05 was considered statistically significant.

## Result

**Fig. 1:**
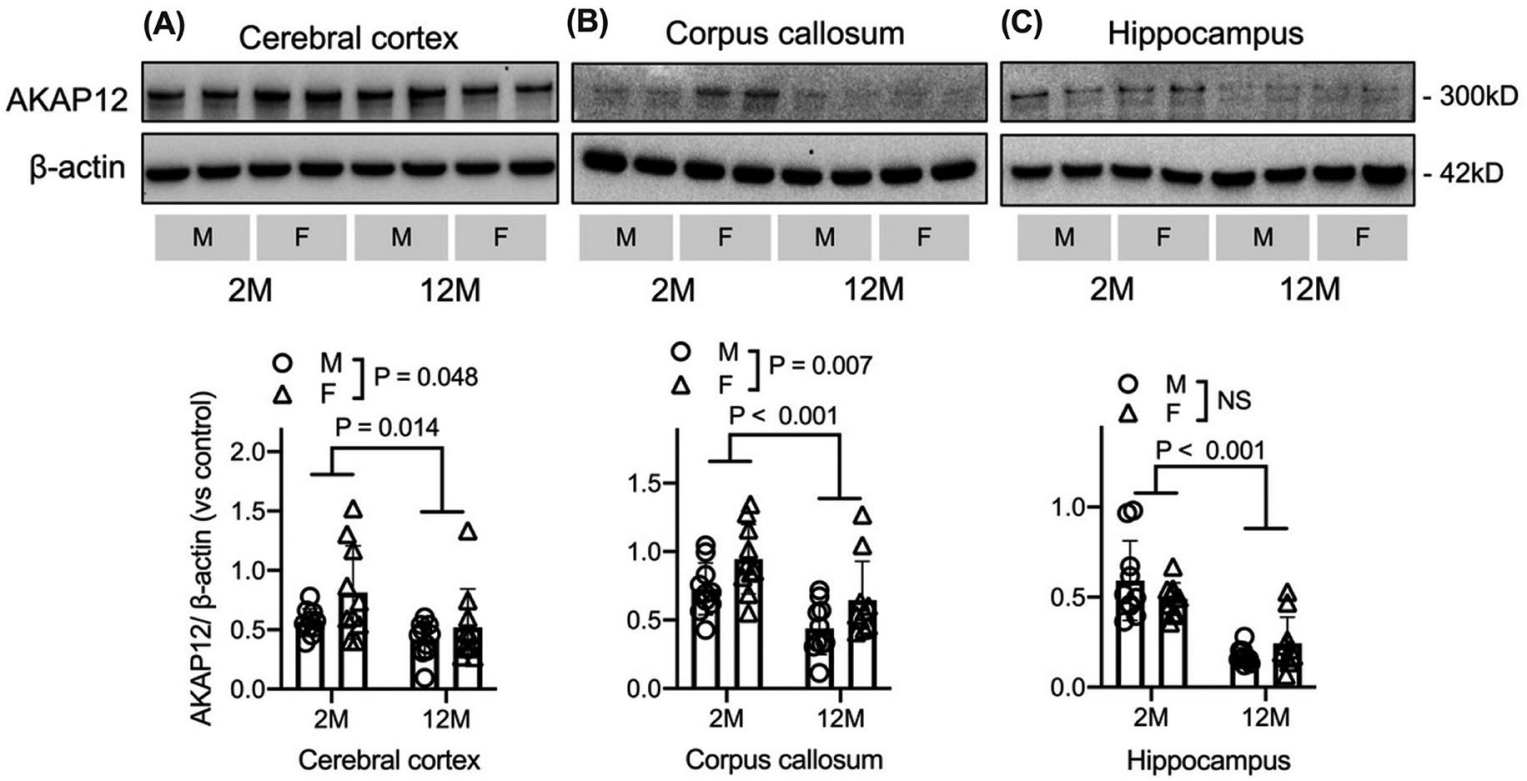
AKAP12 expression in C57BL/6J mice: Male and female C57BL/6J mice (2- and 12-month-old) underwent evaluation. Tissue samples of cerebral cortex (**A**), corpus callosum (**B**) and hippocampus (**C**) were used for this experiment. AKAP12 expression decreased in middle-aged mouse brains compared to young mouse brains in all examined regions. AKAP12 was more abundantly expressed in female mice than in male mice, particularly in the cerebral cortex and corpus callosum. In the level of AKAP12 expression in the cerebral cortex (**A**), no significant Sex x Age interaction was observed (F (1, 36) = 0.73, *P* = 0.40). The expression level was significantly higher in female mice compared to males (F (1, 36) = 4.18, *P* = 0.048), and in 2-month-old mice compared to 12-month-olds (F (1, 36) = 6.654, *P* = 0.014). In the level of AKAP12 expression in the corpus callosum (**B**), no significant Sex x Age interaction was observed (F (1, 36) = 0.005, *P* = 0.95). The expression level was significantly higher in female mice compared to males (F (1, 36) = 8.32, *P* = 0.007), and in 2-month-old mice compared to 12-month-olds (F (1, 36) = 16.00, *P* < 0.001). In the level of AKAP12 expression in the hippocampus (**C**), neither Sex x Age interaction nor the difference between male and female mice was significant (Interaction: F (1, 36) = 3.67, *P* = 0.063; Sex: F (1, 36) = 0.12, *P* = 0.73). The expression level was significantly higher in 2-month-old mice compared to 12-month-olds (F (1, 36) = 55.19, *P* < 0.001). Two-way ANOVA was used for the analysis, with *N* = 10 for each group

Because this study focuses on sex differences in AKAP12-related behavior in mice (and because our previous study shows that AKAP12 may decrease with age [10]), we first used normal C57BL/6J mice to investigate whether there are age and/or sex differences in AKAP12 expression levels. We collected brains from 2- and 12-month-old male and female mice. Brain samples from the cerebral cortex, corpus callosum and hippocampus were separately subjected to Western blot analysis to measure AKAP12 levels. As shown in Fig. 1, AKAP12 levels decreased with age in all three brain regions. In addition, a sex difference in AKAP12 levels was observed in the cerebral cortex and corpus callosum, with slightly but significantly higher expression in female mice.

**Fig. 2:**
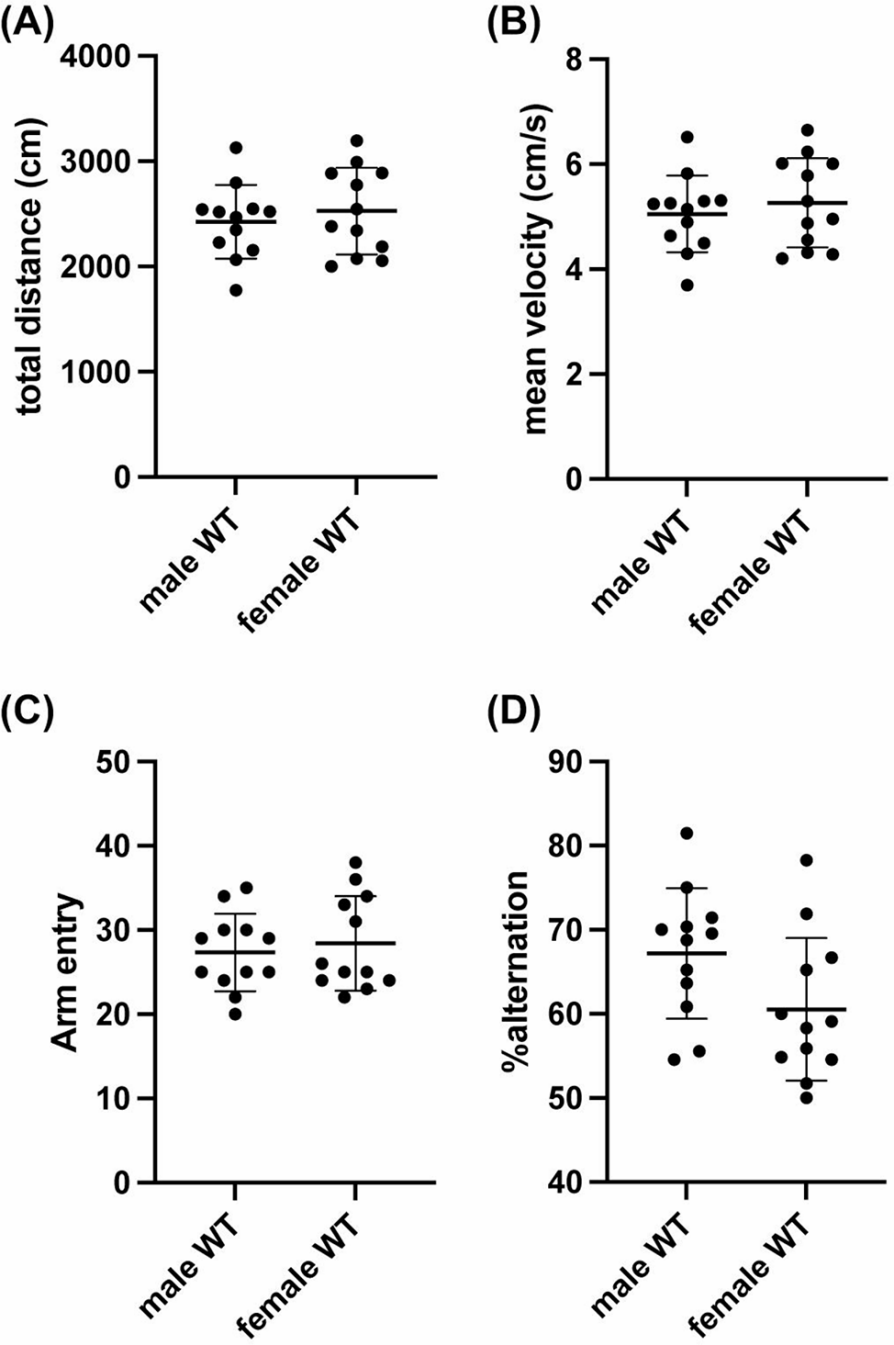
Working memory in middle-aged C57BL/6J mice: Middle-aged male and female mice underwent evaluation using the spontaneous Y-maze test to examine their cognitive function. (**A**-**B**) During the Y-maze test, there were no significant differences between male and female mice in either movement distance (*P* = 0.51, Unpaired t-test) or mean velocity (*P* = 0.51, Unpaired t-test). (**C**) No significant differences were observed in the total number of arm entries between male and female mice, indicating comparable locomotor activity (*P* = 0.74. Mann-Whitney test). (**D**) There were no differences in the alternation ratio (index of working memory) (*P* = 0.057, Unpaired t-test). *N* = 12 for each group

**Fig. 3:**
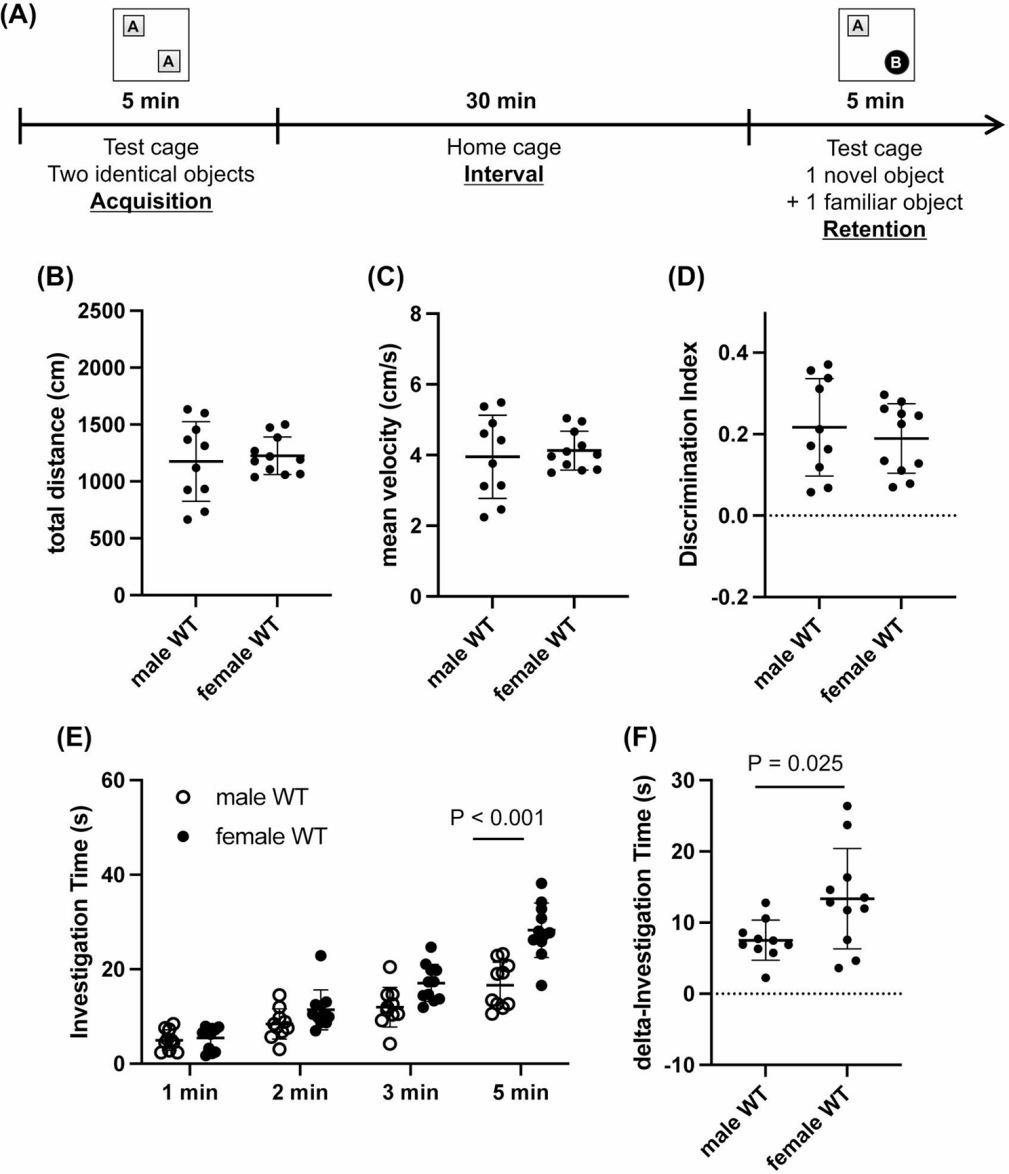
Short-term memory and exploratory behavior in middle-aged C57BL/6J mice: (**A**) Experimental timeline for the Novel Object Recognition Test. (**B**-**C**) During the NORT, there were no significant differences between male and female mice in either movement distance (*P* = 0.67, Unpaired t-test) and mean velocity (*P* = 0.66, Unpaired t-test). (**D**) The discrimination index (DI) was calculated using the formula: DI = (the time spent on the novel object) / (the total time spent on both objects) − 0.5. No significant differences were observed in the DI between males and females (*P* = 0.55, Unpaired t-test). (**E**) The total investigation time in the retention phase was significantly longer in female mice compared to male mice (Sex x Time interaction: F (3, 76) = 7.38, *P* < 0.001; Sex: F (1, 76) = 32.9, *P* < 0.001). (**F**) The delta investigation time was determined by subtracting the total investigation time in the acquisition phase from the total time spent in the retention phase. The delta investigation time was extended in female mice compared to males (*P* = 0.025, Unpaired t-test). *N* = 10 for male WT and *N* = 11 for female WT

We next examined whether there were sex differences in cognitive function in middle-aged mice using two widely used tests of cognitive function: the spontaneous Y-maze test and the novel object recognition test (NORT). In the Y-maze test, there were no significant differences between male and female mice in either movement distance, mean velocity, number of arm entries (an index of mouse activity) or percentage of alternations (an index of working memory) (Fig. 2a-d). Similarly, in the NORT experiments, no sex difference was observed in movement distance, mean velocity, and Discrimination Index (an index of short-term memory) (Fig. 3a-d). However, during the retention phase, female mice tended to spend more time near the objects compared to male mice (Fig. 3e), suggesting a difference in exploratory behavior between the sexes. In addition, delta exploration time (the difference in exploration time between the memory acquisition and retention phases) also showed that female mice exhibited greater exploratory behavior (Fig. 3f).

**Fig. 4:**
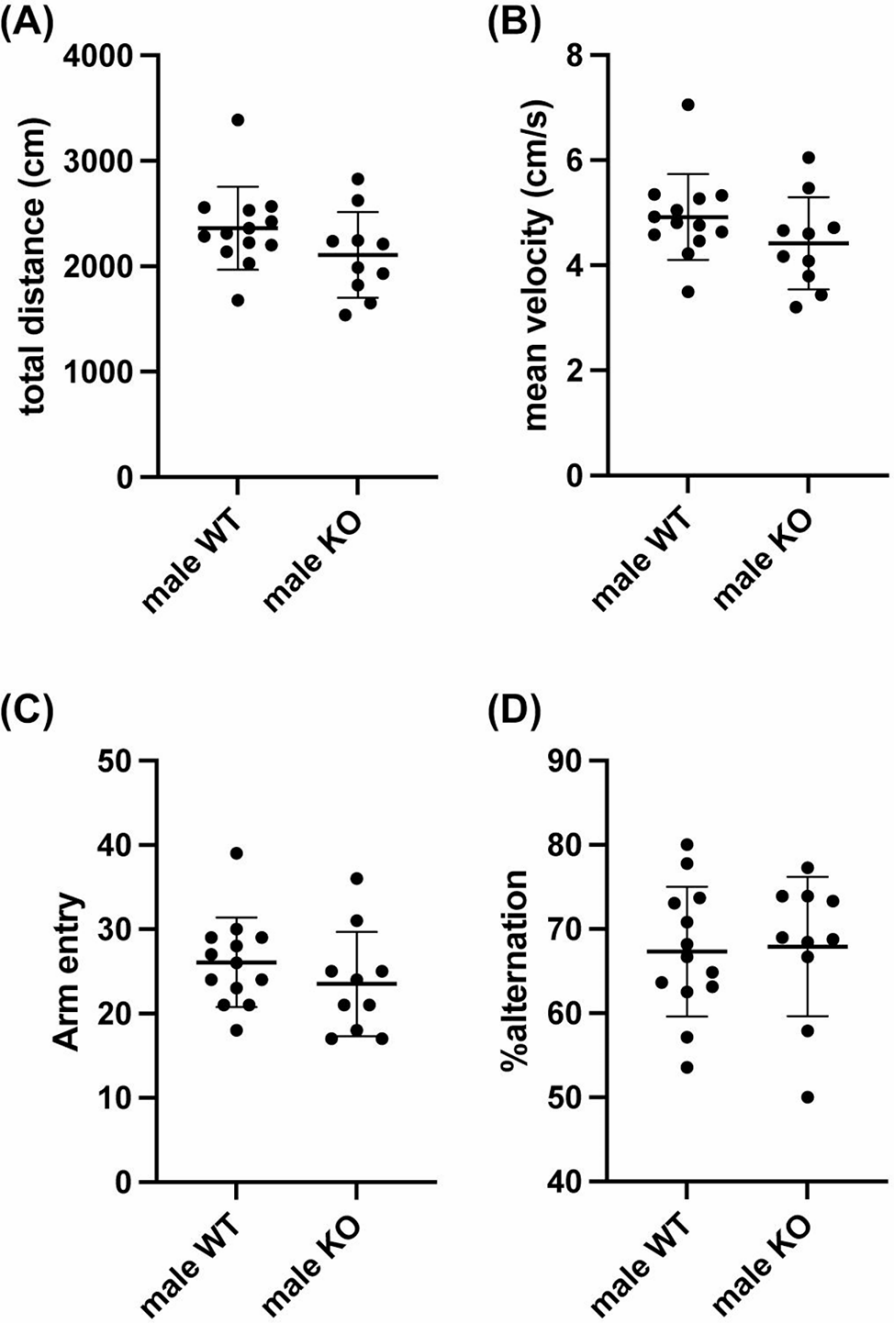
Working memory in middle-aged male WT and Akap12 KO mice: Both male WT and Akap12 KO mice were evaluated using the spontaneous Y-maze test. (**A**-**B**) During the Y-maze test, there were no significant differences between male WT and male KO mice in either movement distance (*P* = 0.15, Unpaired t-test) or mean velocity (*P* = 0.17, Unpaired t-test). In addition, there were no significant differences in both the number of the arm entry (**C**) and the alternation ratio (**D**) between the male WT and Akap12 KO groups (Arm entry: *P* = 0.30, Unpaired t-test; Alternation: *P* = 0.86, Unpaired t-test). *N* = 13 for male WT and *N* = 10 for male KO

**Fig. 5:**
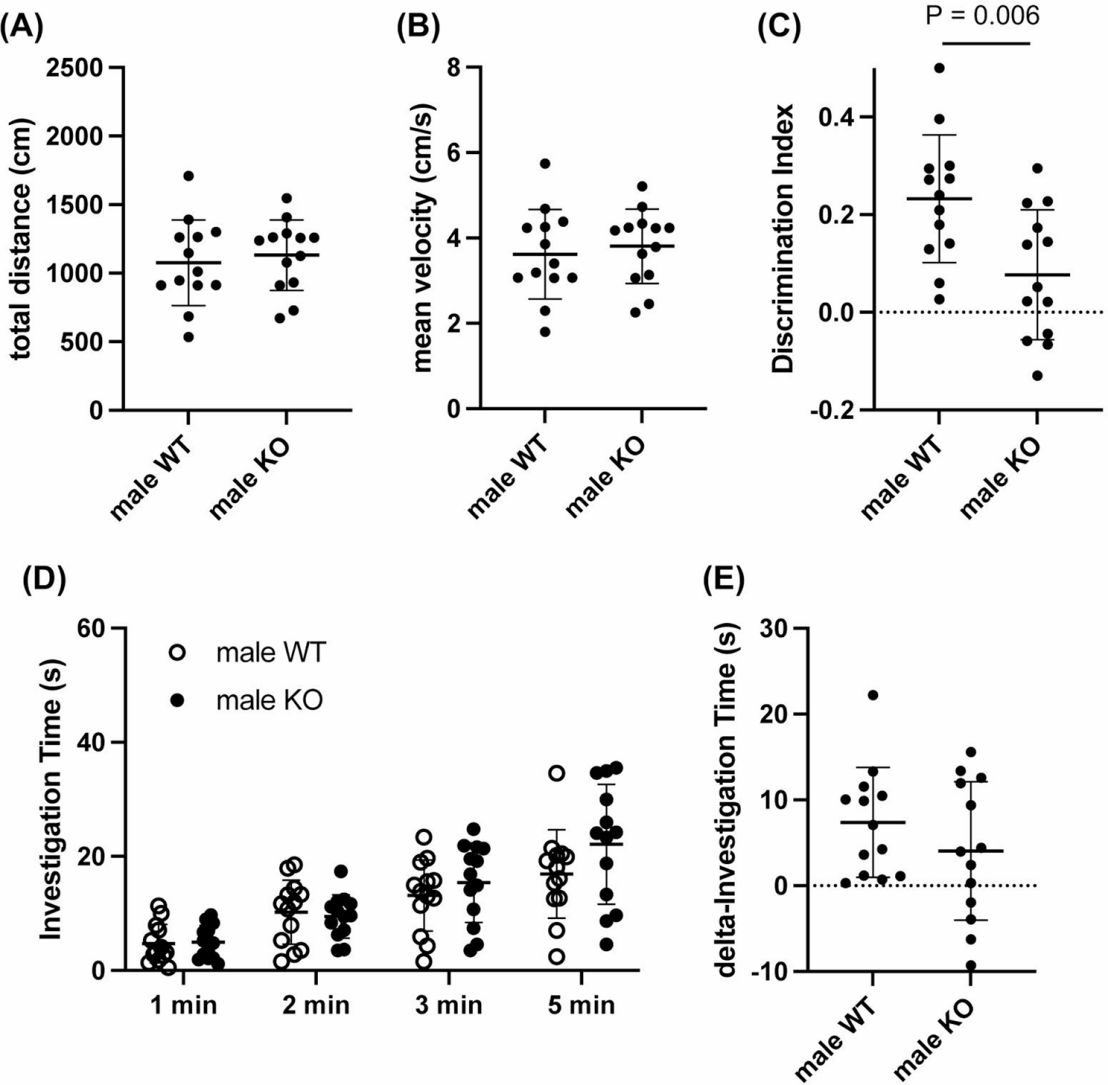
Short-term memory and exploratory behavior in middle-aged male WT and Akap12 KO mice: (**A**-**B**) During the NORT, there were no significant differences between male WT and male KO mice in either movement distance (*P* = 0.63, Unpaired t-test) and mean velocity (*P* = 0.62, Unpaired t-test). (**C**) The discrimination index was significantly lower in the male Akap12 KO mice (*P* = 0.0060, Unpaired t-test). (**D**) There was no significant difference in the total investigation time between the male WT and Akap12 KO groups (Genotype x Time interaction: F (3, 96) = 1.11, *P* = 0.35; Genotype: F (1, 96) = 1.97, *P* = 0.16, 2-way ANOVA). (**E**) There was no significant difference in the delta investigation time between the male WT and Akap12 KO groups (*P* = 0.26, Unpaired t-test). *N* = 13 per group

**Fig. 6:**
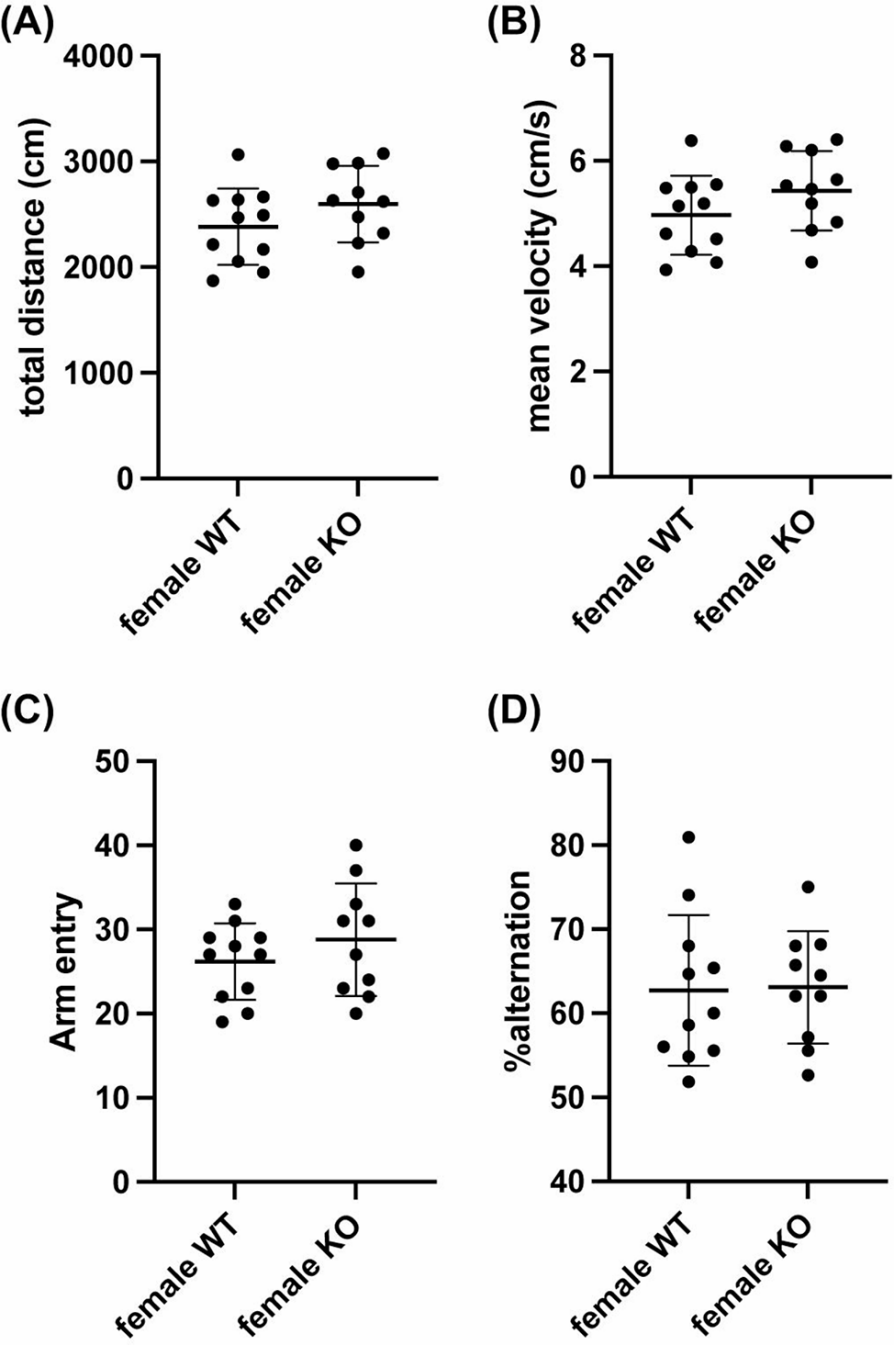
Working memory in middle-aged female WT and Akap12 KO mice: Both female WT and Akap12 KO mice were evaluated using the spontaneous Y-maze test. (**A**-**B**) During the Y-maze test, there were no significant differences between female WT and female KO mice in either movement distance (*P* = 0.19, Unpaired t-test) or mean velocity (*P* = 0.18, Unpaired t-test). In addition, there were no significant differences in both the number of the arm entry (**C**) and the alternation ratio (**D**) between the female WT and Akap12 KO groups (Arm entry: *P* = 0.30, Unpaired t-test; Alternation: *P* = 0.92, Unpaired t-test). *N* = 11 for female WT and *N* = 10 for female Akap12 KO

**Fig. 7:**
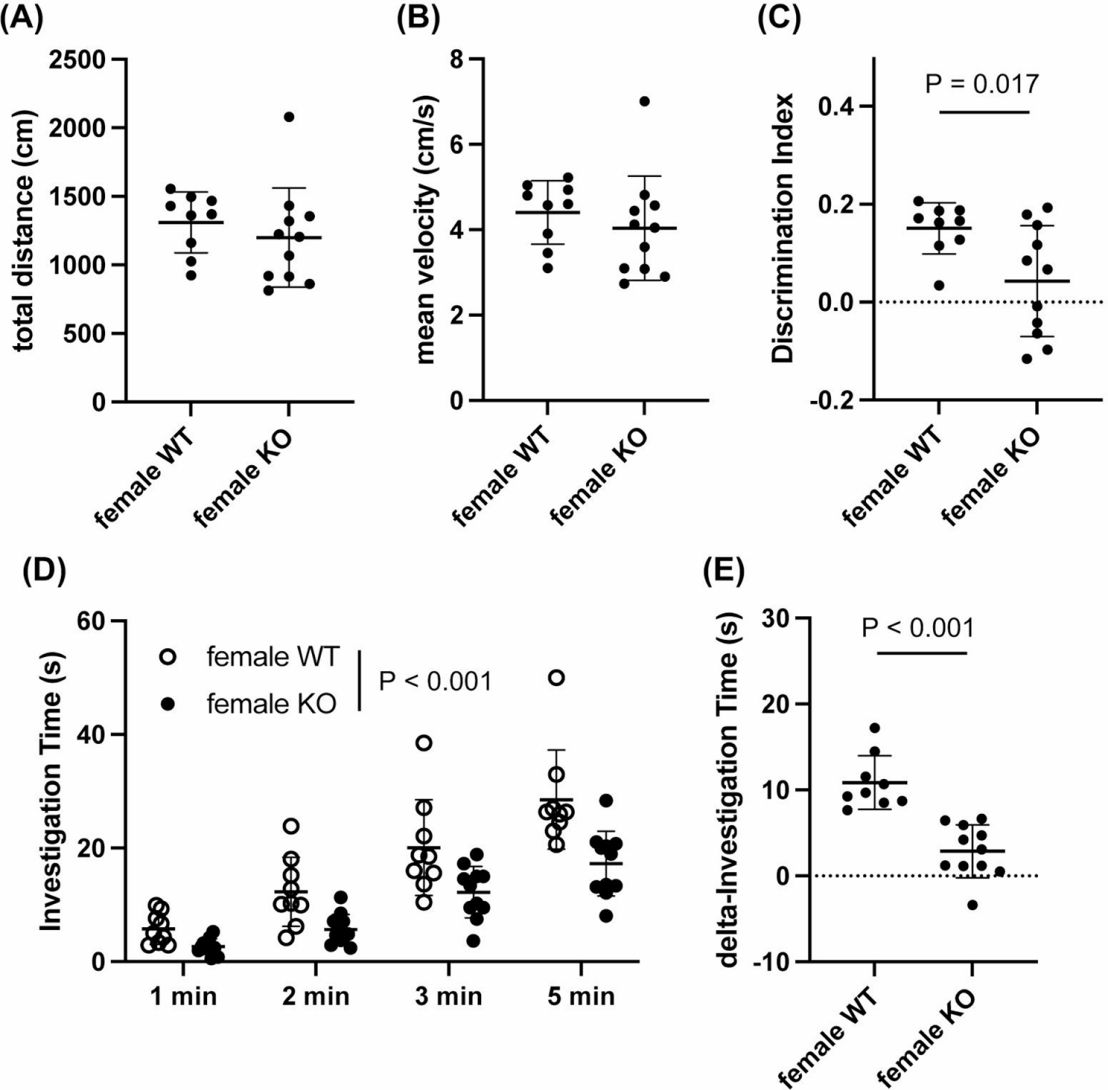
Short-term memory and exploratory behavior in middle-aged female WT and Akap12 KO mice: (**A**-**B**) During the NORT, there were no significant differences between female WT and female KO mice in either movement distance (*P* = 0.43, Unpaired t-test) and mean velocity (*P* = 0.44, Unpaired t-test). (**C**) The discrimination index was significantly lower in the female Akap12 KO mice (*P* = 0.017, Unpaired t-test). (**D**) There was a significant difference in the total investigation time in the retention phase between the female WT and Akap12 KO groups (Genotype x Time interaction: F (3, 72) = 1.88, *P* = 0.14; Genotype: F (1, 72) = 34.57, *P* < 0.001, 2-way ANOVA). (**E**) There was a significant difference in the delta investigation time between the female WT and Akap12 KO groups (*P* < 0.001, Unpaired t-test). *N* = 9 for female WT and *N* = 11 for female Akap12 KO

While AKAP12 levels decreased with age, middle-aged mice still showed some AKAP12 expression, with higher levels in female mice. Therefore, we finally investigated whether AKAP12 deficiency would affect cognitive and exploratory behavior in middle-aged mice. In male mice, Akap12 KO mice showed a similar movement distance, mean velocity, number of arm entries, and percentage of spontaneous alternation in the Y-maze test compared to wild-type (WT) mice (Fig. 4a-d). On the other hand, Akap12 KO mice showed a lower discrimination index score in the NORT, without showing any dysfunction in movement behavior (Fig. 5a-c), suggesting that AKAP12 may play a role in short-term memory in middle-aged male mice. There were no significant differences in investigation time during the memory retention phase and delta investigation time between WT and Akap12 KO male mice (Fig. 5d-e). In middle-aged female mice, Akap12 KO mice also showed a similar movement distance, mean velocity, number of arm entries and percentage of spontaneous alternation in the Y-maze test compared to WT mice (Fig. 6a-d). In addition, similar to the experiments using male mice, female Akap12 KO mice showed a lower discrimination index score in the NORT, without showing any dysfunction in movement behavior (Fig. 7a-c), suggesting that AKAP12 may play a role in short-term memory also in middle-aged female mice. However, in contrast to the comparison between male WT and Akap12 KO mice, exploratory behavior was suppressed in female Akap12 KO mice compared to WT mice in the NORT (Fig. 7d-e).

## Discussion

In this study, we aimed to investigate the role of AKAP12 in cognitive and exploratory behaviors in middle-aged mice, focusing on potential sex differences. Our results showed that (i) AKAP12 levels decreased with age in the cerebral cortex, corpus callosum and hippocampus, (ii) AKAP12 expression was higher in the cerebral cortex and corpus callosum of female mice compared to males, (iii) no sex differences in cognitive function were observed in the Y-maze and the NORT, but female mice showed greater exploratory behavior during the NORT, and (iv) AKAP12 deficiency impaired short-term memory in both sexes, but suppressed exploratory behavior only in female mice.

Sex differences in brain function and pathology are important areas of research as they may influence both normal brain function and disease outcomes [20, 21]. Our behavioral assessments using the spontaneous Y-maze test and NORT revealed no significant sex differences in working memory and short-term memory, consistent with previous studies in younger mice [22]. However, female mice showed greater exploratory behavior in the NORT, consistent with reports of a female advantage in object feature memory in several species, including humans [23] and rats [24]. The advantage in discriminating similar objects has also been reported in female mice [25], and our results may highlight the sex difference in object investigation/seeking behavior. Notably, our study is the first to demonstrate sex differences in AKAP12 expression in the brain. The higher AKAP12 expression in the cerebral cortex and corpus callosum of female mice may contribute to the more pronounced effects of AKAP12 deficiency on exploratory behavior, as evidenced by the suppression of exploratory behavior in female Akap12 KO mice during the NORT. The frontal cortex, which is active during exploration [26], is likely to play a role in this context.

Our study also provides insight into the role of AKAP12 in cognitive function. AKAP12 deficiency reduced discrimination indices in both sexes in the NORT, suggesting that AKAP12 plays a role in maintaining short-term memory in middle-aged mice. This decline may be associated with reduced AKAP12 levels in the hippocampus, a region essential for the retention and transfer of short-term memories to long-term storage [27]. Previous research has shown that AKAP12-deficient mice have impairments in PKA-dependent and β2-adrenergic receptor-mediated hippocampal synaptic plasticity, leading to long-term memory deficits [11]. Thus, the age-related decrease in hippocampal AKAP12 levels likely contributes to the observed decline in short-term memory in middle-aged mice. Interestingly, while young Akap12 KO mice showed reduced spatial memory in the Y-maze test [12], no significant differences were found in the same Y-maze test between middle-aged Akap12 KO and WT mice in our current study. This lack of difference may be due to the general decline in spatial memory with age, which affects both KO and WT mice.

Previous studies have primarily focused on young male mice to investigate the role of AKAP12 in the CNS, and therefore, our study provides valuable insights into how AKAP12 contributes to sex-related brain functions in middle-aged mice. However, several caveats and limitations should be considered. First, we only assessed exploratory behavior by the duration of investigation time during the NORT. Future studies should include additional approaches to investigate the role of AKAP12 in exploratory behavior. Second, while the Y-maze and NORT are well validated methods [28, 29], other cognitive tests such as the water maze, radial arm maze, and passive avoidance test should be used to more comprehensively examine the role of AKAP12 in cognitive function. In addition, because our NORT protocol only assesses short-term memory, other protocols that assess long-term memory may need to be considered to gain further insight into the role of AKAP12 in cognitive function [11]. Third, our current study does not examine the mechanism by which AKAP12 deficiency leads to cognitive dysfunction in middle-aged mice. It has been observed that Akap12 KO mice have decreased fertility along with prostate hyperplasia [13]. Therefore, it is possible that Akap12 KO mice show an imbalance in hormone levels that may affect cognitive function, since reproductive tissues are affected in Akap12 KO mice and the beneficial effects of sex hormones on memory function are well documented [30–32]. In addition, many proteins change during aging and it is possible that AKAP12 deficiency affects these age-related changes in protein expression levels because AKAP12 regulates multiple intracellular signaling pathways. Elucidating the cellular and molecular mechanisms of AKAP12 roles in cognitive function would deepen our understanding of age- and sex-related cognitive function/dysfunction. Finally, cognitive decline/dysfunction may result from brain pathology, such as stroke and vascular dementia, and therefore, future research should investigate the role of AKAP12 in the pathological mechanisms of brain disease. Accumulating evidence suggests that AKAP12 plays protective roles in the CNS, including maintaining BBB integrity and supporting myelination under ischemic conditions [10, 12, 33]. Thus, AKAP12 may serve as a novel therapeutic target for cognitive decline associated with stroke or other CNS diseases.

In conclusion, our study shows that AKAP12 expression in the brain differs between the sexes and declines with age. These expression differences may influence behavior, particularly exploratory behavior in females. AKAP12 is also important for the maintenance of short-term memory in middle-aged mice, highlighting its important role in cognitive function and behavior. Future research is warranted to explore the therapeutic potential of AKAP12 in sex-related brain diseases.

## Electronic supplementary material

Below is the link to the electronic supplementary material.

Supplementary Material 1

## Data Availability

Raw data of western blot and behavior experiments will be available upon reasonable request.
