# Supplementary material for: Sex differences in the role of AKAP12 in behavioral function of middle-aged mice
Source: Biol Sex Differ. 2024 Nov 21;15:93. doi: 10.1186/s13293-024-00670-8 (PMC11580627; doi:10.1186/s13293-024-00670-8)
Supplement: Supplementary file 1 — Supplementary Material 1 [file 13293_2024_670_MOESM1_ESM.docx]

**Supplementary information**

**Sex differences in the role of AKAP12 in behavioral function of middle-aged mice**

Hidehiro Ishikawa^1, 2, 8^, Shintaro Kimura^1, 3, 8^, Hajime Takase^1, 4^, Maximillian Borlongan^1^, Norito Fukuda^1^, Tomonori Hoshino^1^, Gen Hamanaka^1^, Ji Hyun Park^1^, Akihiro Shindo^2^, Kyu-Won Kim^5^, Irwin H Gelman^6^, Josephine Lok^1, 7^, Eng H. Lo^1^, Ken Arai^1, *^

^1^ Neuroprotection Research Laboratories, Departments of Radiology and Neurology, Massachusetts General Hospital and Harvard Medical School, Charlestown, MA, USA

^2^ Department of Neurology, Mie University Graduate School of Medicine, 2-174 Edobashi, Tsu, Mie, Japan

^3^ Life Science Research Center, Gifu University, Gifu, Japan

^4^ YCU Center for Novel and Exploratory Clinical Trials (Y-NEXT), Yokohama City University Hospital, Yokohama, Japan

^5^ College of Pharmacy and Research Institute of Pharmaceutical Sciences, Seoul National University, Seoul 08826, Republic of Korea

^6^ Department of Cancer Genetics and Genomics, Roswell Park Comprehensive Cancer Center, Buffalo, NY, USA

^7^ Pediatric Critical Care Medicine, Department of Pediatrics, Massachusetts General Hospital and Harvard Medical School, Boston, MA, USA

^8^ These authors equally contributed to this work.

***Corresponding author**

Ken Arai, Ph.D.

karai@partners.org

149 Thirteenth Street, Room 2401 Charlestown, Massachusetts 02129-2000, USA

TEL: 617-724-9530, FAX: 617-726-7830


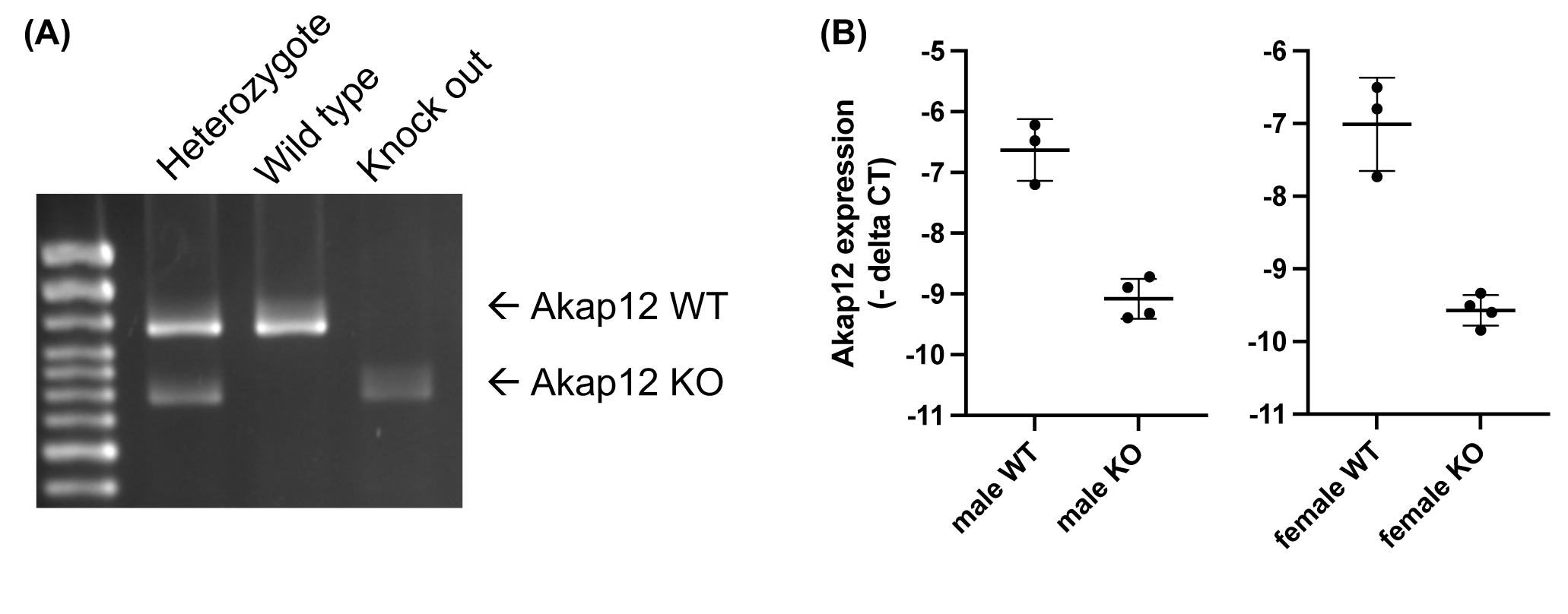


**Supplementary Figure S1. Validation of our Akap12 KO mouse line:** **(A)** A representative image of genotyping PCR for identification of wild-type, heterozygous and homozygous Akap12 knockout mice. **(B)** RT-qPCR using brain samples from male/female WT and KO mice. Total RNA was reverse transcribed to cDNA using the QuantiTect Reverse Transcription Kit (QIAGEN). RT-qPCR analysis was performed using QuantStudio 3 (Applied Biosystems, Waltham, MA, USA) with TaqMan Fast Advanced Master Mix (Thermo Fisher Scientific, Waltham, MA, USA). Expression levels for all transcripts were normalized to that of Hprt. All TaqMan gene expression assays were purchased from Applied Biosystems: Akap12 (Mm00513511_m1) and Hprt (Mm01545399_m1, internal control).
